# Overnutrition induced metabolic dysregulation and partially decreased semen quality in young beef bulls

**DOI:** 10.1093/jas/skag004

**Published:** 2026-01-12

**Authors:** Pedro L P Fontes, Dylan B Davis, Lucas Melo-Gonçalves, Samir Burato, Molly S Smith, Alexander Stelzleni, Francis F Fluharty, R Lawton Stewart, Jr., Reinaldo F Cooke, John J Bromfield, Alexandra Else-Keller, Karl Kerns, Lew Strickland, Saulo M Zoca

**Affiliations:** Department of Animal and Dairy Science, University of Georgia, Athens, GA 30602; Department of Animal and Dairy Science, University of Georgia, Athens, GA 30602; Department of Animal and Dairy Science, University of Georgia, Athens, GA 30602; Department of Animal and Dairy Science, University of Georgia, Athens, GA 30602; Department of Animal and Dairy Science, University of Georgia, Athens, GA 30602; Department of Animal and Dairy Science, University of Georgia, Athens, GA 30602; Department of Animal and Dairy Science, University of Georgia, Athens, GA 30602; Department of Animal and Dairy Science, University of Georgia, Athens, GA 30602; Department of Animal Science, Texas A&M University, College Station, TX 77843; Department of Animal Sciences, University of Florida, Gainesville, FL 32611; Department of Animal Science, Iowa State University, Aimes, IA 50011; Interdepartamental Genetics and Genomics, Iowa State University, Aimes, IA 50011; Department of Animal Science, Iowa State University, Aimes, IA 50011; Department of Animal Science, University of Tennessee, Knoxville, TN 37996; Department of Animal Science, University of Tennessee, Knoxville, TN 37996

**Keywords:** beef, diet, male fertility, semen, sire

## Abstract

The objective of this study was to characterize the systemic metabolic response to overnutrition in young bulls and to evaluate the effects of overnutrition on semen characteristics. Half-sibling yearling beef bulls (*n* = 44) were utilized in a completely randomized design, where bulls were randomly assigned 1 of 2 dietary treatments (*n* = 4 pens/treatment): 1) Moderate Gain (MG): diet formulated to promote an average daily gain of 1.2 kg/day, or 2) High Gain (HG): diet formulated to promote an average daily gain of 1.8 kg/day. Bulls were housed in a feedlot facility equipped with an automated individual feed intake monitoring system and fed their respective diets for 114 days. Body weight, carcass ultrasonography, and blood samples were collected on days 0, 36, 76, and 114. Blood samples were utilized to determine circulating concentrations of cholesterol, triglycerides, glucose, insulin, leptin, low-density lipoprotein, non-esterified fatty acids (NEFA), beta-hydroxybutyrate (BHB), testosterone, and haptoglobin. Serial semen samples were collected at the end of the feeding period (days 109, 111, and 114) and analyzed using computer assisted sperm analysis and image-based flow cytometry. Body weight and subcutaneous backfat thickness were greater (*P *< 0.01) at the end of the feeding period in HG compared with MG bulls. Similar results were observed for circulating concentrations of glucose, insulin, leptin, cholesterol, and low-density lipoproteins (*P *≤ 0.02). Alternatively, circulating concentrations of NEFA and BHB were decreased (*P *< 0.01) in HG bulls at the end of the feeding period. Bulls exposed to the HG diet had greater (*P *< 0.01) insulin resistance at the end of the feeding period based on insulin: glucose ratio and revised quantitative insulin sensitivity check index (RQUICKI). HG bulls had greater (*P *= 0.02) plasma haptoglobin compared with MG bulls, whereas testosterone concentrations were similar (*P *= 0.69). Bulls exposed to the HG diet tended to have decreased (*P *≤ 0.09) total and progressive motility compared with MG bulls. Moreover, the proportion of sperm with partially damaged acrosomes tended (*P *= 0.09) to be increased and the proportion of sperm with intact plasma membrane tended to be reduced (*P *≤ 0.10) in HG bulls compared with MG bulls. In summary, HG dietary treatment promoted an obese-like metabolic profile that increased insulin resistance and circulating haptoglobin, and resulted in a subtle decrease in semen quality.

## Teaser

Male overnutrition induces increased adiposity, insulin resistance, and negatively impacted semen quality.

## Abbreviations:

BHB, beta-hydroxybutyrate; CASA, computer assisted sperm analysis; ddH2O, double-distilled water; HG, high gain; IVC, in vitro capacitation; IVP, in vitro embryo production; LDL, low-density lipoproteins; LMA, longissimus dorsi area; MG, moderate gain; NEFA, non-esterified fatty acids; PI, propidium iodide; PBS, phosphate buffer saline; PNA, peanut agglutinin; RQUICKI, revised quantitative insulin sensitivity check index

## Introduction

Male obesity in humans is a global health concern, with its prevalence steadily increasing alongside rising rates of infertility. Approximately 45% of human infertility cases are attributed to the male partner, and growing evidence indicates that obesity is a major contributor to this trend (Craig et al.., 2017). Obesity induces a cascade of metabolic and endocrine disturbances, including altered insulin sensitivity, systemic inflammation, and oxidative stress (Reaven, 1988; Kahn et al., 2006; Campbell et al., 2015), all of which negatively impact testicular function and spermatogenesis. Beyond its effects on testicular function, obesity also negatively impacts spermiogram parameters in humans, such as sperm motility and morphology, which are critical for fertilization success and embryo development (Mitchell et al., 2011; Craig et al., 2017). Male overnutrition is commonly observed in the beef cattle industry, as beef cattle producers generally prioritize sires with high rates of body weight gain during growth and development (Smith et al., 2025; Fontes et al., 2025). In fact, when selecting herd sires, commercial producers often prioritize genetic traits associated with growth over feed efficiency traits, such as feed-to-gain ratio or residual feed intake (McDonald et al., 2010; Oosthuizen et al., 2018). These preferences drive seedstock producers to expose young bulls to diets that promote rapid growth and fat deposition to achieve this desired marketable phenotype. Notably, such diets result in levels of body weight gain and adiposity that resemble those observed in feedlot steers (Marques et al., 2017; Pancini et al., 2020; Smith et al., 2025) and have been associated with suboptimal bull fertility (Seekford et al., 2023; Fontes et al., 2025).

Observational studies reported that increased body adiposity in young bulls was associated with an increased percentage of morphologically abnormal sperm. Moreover, increased adiposity in bulls is associated with a greater percentage of bulls failing their first breeding soundness examinations (Barth and Waldner, 2002; Smith et al., 2025). Experimentally inducing high adiposity in bulls using calorically dense diets also demonstrated a negative impact on traditional spermiogram outcomes. Bulls exposed to high-energy diets for 168 days had decreased sperm motility and a greater percentage of morphologically abnormal cells compared with bulls fed a less caloric diet (Coulter and Kozub 1984; Coulter et al., 1997). Yet, most studies evaluating the negative impact of high-energy diets on bull fertility were limited to traditional spermiogram parameters, such as motility and morphology evaluated chute-side (Hopkins and Spitzer, 1997; Koziol and Armstrong, 2018). Moreover, systemic metabolic responses and their relationship with semen characteristics have not been thoroughly described in bulls. We hypothesized that bulls exposed to overnutrition have increased adiposity, altered systemic markers that were previously associated with obesity in other species, and subtle decreases in semen quality. Therefore, the objective of this study was to characterize the impact of overnutrition on systemic metabolic responses and semen characteristics in young beef bulls. Specifically, we evaluated how exposure to a high-energy diet al.ers systemic metabolic indicators and semen quality using advanced semen analyses to capture the effects of overnutrition beyond conventional spermiogram parameters.

## Materials and Methods

### Animals and experimental design

All bulls were handled in accordance with procedures approved by the University of Georgia’s Institutional Animal Care and Use Committee (Animal Use Protocol # A2022 01-009-Y3-A0).

All the experimental procedures were performed at the University of Georgia’s Eatonton Beef Research Unit. A total of 44 yearling Angus bulls (age: 417.4 ± 5.35 days; body weight: 411.4 ± 44.36 kg; body condition score 5.4 ± 0.57; mean ± standard deviation) originated from the same herd were utilized in the present study. To minimize age and genetic variation between individuals, all bulls enrolled in the study were half-siblings and sired by the same bull through fixed-time artificial insemination. Bulls were housed in eight pens (four pens per treatment) within a feedlot facility equipped with an automated bunk system (Smartfeed Pro; C-Lock Inc., Rapid City, SD) that allowed for individual feed intake assessments throughout the study. Bulls were brought into the feedlot facility prior to the beginning of the study for a 7-day acclimation period (day -7). During the adaptation period, all bulls were fed a common diet, which was later used in the Moderate Gain (MG) treatment during the experimental feeding regimen. After the period in which bulls were adapted to the facilities, bulls were stratified by age, body weight, and body condition scores and randomly assigned 1 of 2 dietary treatments (day 0): 1) Moderate Gain (MG): diet formulated to promote an average daily gain of 1.22 kg/day, or 2) High Gain (HG): diet formulated to promote an average daily gain of 1.81 kg/day. Diet ingredients were similar between diets, but dietary treatments varied on the rate of inclusion of each ingredient and daily feed intake allowance (Supplementary Table S1—see online supplementary material). At the beginning of the feeding scheme, bulls in both treatments received their respective diets ad libitum, however, to guarantee the proposed targeted average daily gain for the MG treatment, daily intake was adjusted every 14 days in a step-down program to achieve a daily feed intake of 28.2 kCal/kg of body weight (NASEM, 2016). Bulls in the HG treatment had ad libitum access to their diet throughout the feeding period. Diet samples were collected biweekly, combined, and analyzed for nutritive value (Supplementary Table S1—see online supplementary material). The dietary regimen was superimposed for a total of 114 days.

Initial (day 0) and final (day 114) body weights were evaluated based on two independent assessments within a 24 ± 4 h period. Moreover, body weight was determined on days 36 and 76, and body weight estimates were utilized to monitor average daily gain and adjust feed intake allowance of bulls enrolled in the MG treatment. Body composition in response to dietary treatments were evaluated using carcass ultrasonography (3.5 mHz linear array transducer, Aloka 500 V; Corimetrics Medical Systems Inc., Wallingford, CT, USA; Emenheiser et al., 2014) on days 0, 36, 76, and 114. Subcutaneous backfat, intramuscular fat, and longissimus dorsi area were collected and then analyzed using Beef Information Analysis Pro Plus software (Designer Genes USA, Harrison, AR; Detweiler et al., 2019). Scrotal circumference, paired-testis volume, and scrotal skin thickness were collected on days 0 and 114 according to (Byrne et al., 2018). Moreover, scrotal surface temperature was evaluated in defined areas of the scrotum using a FLIR T640 thermal imaging camera (FLIR Systems Inc., Wilsonville, OR, USA) on day 114. While bulls were restricted in the chute, the camera was positioned 1 meter away from the scrotum to capture a caudal image while standardizing imaging distance and angle between bulls. All samples were collected before 10:00, and the order of image collection was alternated between treatments to ensure equal distribution of bulls from both treatments across the image acquisition period. Image analysis was performed using FLIR Tools software (FLIR Systems Inc.), where regions of interest encompassing the scrotal surface were manually defined according to Coulter et al. (1997). Scrotal surface temperature was estimated in the top and bottom portions of the scrotum, as well as over each testis. The top portion represented the dorsal aspect of each testis, whereas the bottom portion was selected approximately 1.5 cm above the ventral bottom of the scrotum (Supplementary Figure S1—see online supplementary material for a colour version of this figure). Scrotal surface temperature gradient was estimated by subtracting the average temperature of the bottom of the scrotum from the average temperature of the top. Serial semen collections were performed at the end of the feeding period. Semen collection and processing procedures are described below.

### Laboratorial analyses of plasma metabolic parameters and insulin resistance

Blood samples were collected via coccygeal venipuncture using 6-mL vacutainer tubes (BD Vacutainer, Becton, Dickinson and Company, NJ) on days 0, 36, 76, and 114. Samples were collected into tubes containing lithium heparin for plasma collection and tubes without anticoagulant for serum collection. Blood samples were immediately placed on ice and centrifuged at 2,500 × g for 20 minutes at 4 °C. Plasma and serum were subsequently transferred into polypropylene tubes and stored at -20 °C until further analyses. All samples were analyzed for plasma concentrations of triglycerides, cholesterol, low-density lipoproteins (LDL), non-esterified fatty acids (NEFA), and beta-hydroxybutyrate (BHB) using the Carysta High Volume Chemistry Analyzer (Zoetis Animal Health, Parsipanny, NJ; Mackey et al., 2024). Moreover, radioimmunoassay was utilized to analyze circulating concentrations of insulin (cat #PI-12K; Millipore Sigma, Burlington, MA; Miqueo et al., 2019) and leptin (cat #XL-85K; Millipore Sigma; Delavaud et al., 2000). Lastly, plasma samples from day 114 were also used to estimate circulating concentrations of total testosterone (cat # 0718910; MP Biomedicals, Santa Ana, CA; Venturini et al., 2025) and haptoglobin according to Cooke and Arthington (2013). All plasma samples were analyzed in duplicates for all assays and intra- and inter-assay coefficient of variation were ≤ 3.13 and ≤ 9.79%, respectively. Insulin resistance was determined using the revised quantitative insulin sensitivity check index (RQUICKI), a methodology previously utilized to estimate insulin sensitivity in ruminants based on circulating concentrations of glucose, NEFA, and insulin (Perseghin et al., 2001; Sousa et al., 2022). The following equation was utilized according to Perseghin et al., (2001): RQUICKI = 1/[log(glucose) + log(insulin) + log(NEFA)], where a lower RQUICKI value indicates increased insulin resistance. Glucose was expressed in mg/dL, insulin in µU/mL, and NEFA in mmol/L, consistent with previous studies validating this index in cattle.

### Semen collection and computer assisted sperm analysis

Serial semen collections were collected at the end of the feeding period using electroejaculation (Pulsator V, Lane Manufacturing Inc., Denver, CO, USA). An initial cleanout collection was performed on day 106, followed by three collections performed on days 109, 111, and 114. Semen samples collected during the cleanout collection were not included in the analyses described below. Semen processing followed previously described methods (Seekford et al., 2023). Briefly, ejaculate volume was assessed upon collection, and sperm concentration was estimated using a densimeter (Animal Reproduction Systems Inc., Ontario, CA, USA). Upon concentration assessment, semen samples were diluted in pre-warmed commercial extender solution (OptiXcell, IMV Technologies, Brooklyn Park, MN, USA) to a final concentration of 50 × 10^6^ sperm/mL. Extended semen was then refrigerated for at least three hours before further processing.

Sperm motility and kinematics were determined using a computer-assisted sperm analysis (CASA; IVOS II, Hamilton Thorne, Beverly, MA, USA) at 37 °C. Samples were loaded in Leja slides (Leja Standard, Count 4-chamber, 20 μm chamber depth) and analyzed in duplicates, where six fields per replicate were analyzed using auto-capture. The CASA program settings designed for bovine semen (Hamilton Thorne, Beverly, MA, USA) were utilized according to Zoca et al., (2020). Response variables from CASA reported herein included total motility (%), progressive motility (%), local motility (%), immotile sperm (%), along with total and progressive composite scores. These composite scores were generated by integrating curvilinear velocity, average path velocity, straight-line velocity, amplitude of lateral head displacement, linearity, and straightness. Analyses were performed under the following instrument settings: temperature at 37 °C; 30 frames per capture, and a capture speed of 60 Hz. Cell detection criteria included a minimum cell size of 5 μm^2^ and a minimum head brightness of 160 units. For motility classification, the following thresholds were utilized: maximum cell travel distance of 15 μm, minimum straightness of 70% and minimum average path velocity of 50 μm/s for progressive motility.

### Image-based flow cytometry

Hoechst 33342 (H33342; Calbiochem, Cat# 382065, San Diego, CA, USA) was reconstituted in double-distilled water (ddH_2_O) to a stock concentration of 18 mM. Propidium iodide (PI; Acros Organics, Cat# AC440300010, Geel, Belgium) was prepared at 1 mg/mL in ddH_2_O. Alexa Fluor™ 647-conjugated peanut agglutinin (PNA-AF647; Invitrogen, Cat# L32460) was reconstituted in ddH_2_O at 0.5 µg/mL. For DNA fragmentation analysis, the Roche In Situ Cell Death Detection Kit, Fluorescein (Cat# 11684795910; Basel, Switzerland) was used per the manufacturer’s instructions.

For membrane and acrosomal assessments, semen samples containing approximately 1 × 10^6^ cells were centrifuged at 110 × g for 5 minutes and resuspended in 100 µL of protein-free, non-capacitating medium containing Hoechst 33342 (1:1000), PI (1:1000), and PNA-AF647 (1:2000). Samples were incubated for 30 minutes in the dark at room temperature, washed by centrifugation, and resuspended in phosphate-buffered saline lacking sodium azide for imaging. Plasma membrane and acrosome integrity were analyzed at 0 and 30 minutes of in vitro capacitation (IVC) as previously described (Zoca et al., 2023). In a separate assay for DNA fragmentation, samples were fixed, permeabilized, and labeled with the fluorescein-conjugated TUNEL reagent as directed by the kit protocol. Hoechst 33342 was included in the last 30 minutes at a 1:1000 dilution before final washing.

Samples were analyzed using a Cytek Amnis ImageStream^X^ Mk II instrument (ISX, Fremont, CA, USA) equipped with a 40× objective and INSPIRE software v3.0. The sheath fluid was a phosphate-buffered solution with a pH of 7.2. Flow core diameter and stream speed were set to 6 µm and 66 mm/s, respectively, and data were collected at a rate of up to 2,000 events per second. For the PNA/PI/Hoechst assay, fluorescence excitation and detection were configured as follows: Hoechst 33342 was excited by a 405 nm laser at 10 mW and detected in Channel 7, PI was excited by a 488 nm laser at 60 mW and detected in Channel 5, and PNA-AF647 was excited by a 642 nm laser at 25 mW and detected in Channel 11. Side scatter (SSC) was detected in Channel 6 using a 785 nm laser set to 2 mW. For the TUNEL assay, Hoechst 33342 was excited at 405 nm (10 mW) and detected in Channel 7, while the fluorescein-labeled TUNEL signal was excited by the 488 nm laser (60 mW) and detected in Channel 2. SSC was detected in Channel 6 using the same 785 nm laser and power setting. SpeedBeads were used to maintain consistent focus, and single-cell gating was performed live during acquisition.

Image data were processed using IDEAS software version 6.4 (Cytek). Focused, single-cell events were identified, and morphometric filters were applied to exclude laterally aligned sperm based on previously established image-based criteria (Kerns et al., 2018). Custom masks were created within IDEAS to quantify probe-specific signal intensity and localization, ensuring consistency across samples.

### Statistical analysis

All data were analyzed as a completely randomized design using the SAS statistical package (SAS Inst. Inc., Cary, NC; Version 9.4) and the pen was considered the experimental unit. All the continuous response variables were analyzed using the MIXED procedure, whereas binary response variables were analyzed using the GLIMMIX procedure. Continuous response variables were analyzed assuming a Gaussian distribution, whereas binary response variables were analyzed using a binary distribution with a logit function. Semen-related response variables (sperm morphology, CASA, and flow cytometry outcomes) were analyzed using the GLIMMIX procedure and a beta distribution statement.

Models used to analyze both continuous and binary response variables included the fixed effect of treatment and the random effect of pen(treatment) and bull(pen). Models were different for semen-related response variables, where each ejaculate was considered a replicate within bull. Therefore, models utilized for semen-related response variables included the fixed effect of treatment and the random effect of pen(treatment), bull(pen), and ejaculate(bull). Temporal changes in growth, body composition, and systemic markers of energy balance were analyzed using repeated measures and an unstructured covariance structure statement. Models for repeated measures analyses included the fixed effects of treatment, time, and their respective interaction. Moreover, models included the random effect of pen(treatment) and pen(bull). Exceptions were also made for the analysis of scrotal circumference, paired testis volume, and scrotal skin thickness. Models for these response variables include their respective initial measurements on day 0 as an independent covariate.

All data was analyzed using Satterthwaite approximation to determine the denominator degrees of freedom for tests of fixed effects. Results are reported as least square means and standard error of the means throughout the manuscript. Significance was set at *P *≤ 0.05 and tendencies were determined if 0.05 < *P *≤ 0.10.

## Results

### Dietary treatments resulted in divergent growth rate and body composition

**Figure 1.**
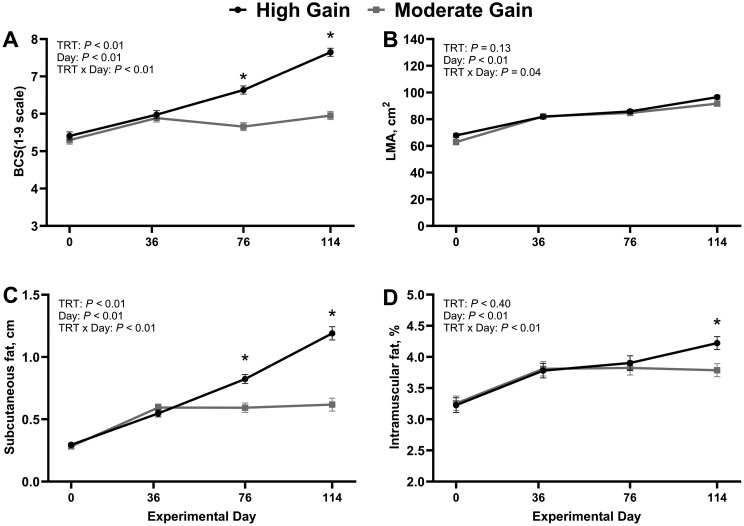
Impact of dietary treatment on temporal changes in bull (*n* = 44) body composition. Moderate Gain (MG): diet formulated to promote an average daily gain of 1.22 kg/d (*n* = 4 pens). High Gain (HG): diet formulated to promote an average daily gain of 1.81 kg/d (*n* = 4 pens). Dietary regimen was superimposed for 114 days. BCS: Body condition score. LMA: Longissimus dorsi muscle area. TRT: treatment. Body composition was determined based on ultrasonography. ^*^Represent statistical differences in least square means between treatment within day (*P* ≤ 0.05).

The impact of treatment on dry matter intake, body weight changes, and feed conversion is summarized in Table 1. Bulls in the HG treatment had greater dry matter intake compared with MG bulls (*P *< 0.01). Body weight was similar between treatments on days 0 and 36 (*P ≥ *0.87) but was greater (*P *< 0.01) in HG compared with MG bulls on both days 76 and 114. Hence, average daily gain was increased (*P *< 0.01) and feed:gain was decreased (*P *< 0.01) in HG compared with MG bulls. Data summarizing temporal changes in body condition score, longissimus dorsi area, subcutaneous backfat thickness, and intramuscular fat are summarized in Figure 1. There was a treatment × day interaction for body condition score, subcutaneous backfat thickness, and intramuscular fat (*P *≤ 0.04), where body condition score and subcutaneous backfat thickness were similar (*P *≥ 0.24) between treatments on days 0 and 36 but were increased (*P *< 0.01) in HG compared with MG bulls on days 76 and 114. Similarly, the percentage of intramuscular fat was not different (*P *≥ 0.62) between treatments on day 0, 36, and 76, but was greater in HG compared with MG bulls on day 114 (*P *< 0.01). There was a treatment × day interaction (*P *= 0.04) for longissimus dorsi area; however, the least square means differences were not significant when comparing treatments within days (*P *≥ 0.34).

**Table 1.** Impact of dietary treatments on bull (*n* = 44) feed intake and body weight gain

	Treatment^1^			
	Moderate Gain	High Gain	SEM	*P*-value
**Dry matter intake, kg/day** ^2^	13.8	17.9	0.59	<0.01
**Body weight**				
** Day 0**	410.3	412.5	9.61	0.87
** Day 36**	543.6	546.2	10.95	0.87
** Day 76**	553.2	622.0	10.83	<0.01
** Day 114**	589.0	682.8	13.04	<0.01
**Average daily gain, kg**	1.6	2.4	0.08	<0.01
**Feed to gain ratio, kg/day**	4.3	3.6	0.16	<0.01

1 Moderate Gain (MG): diet formulated to promote an average daily gain of 1.22 kg/d (*n* = 4 pens), or High Gain (HG): diet formulated to promote an average daily gain of 1.81 kg/d. Dietary regimen was superimposed for 114 days (*n* = 4 pens).

2 Dry matter intake was estimated using an automated feed intake monitoring system (Smartfeed Pro; C-Lock Inc., Rapid City, SD).

### High gain treatment induced an obesity-like metabolic profile, insulin resistance, and increased circulating haptoglobin

**Figure 2.**
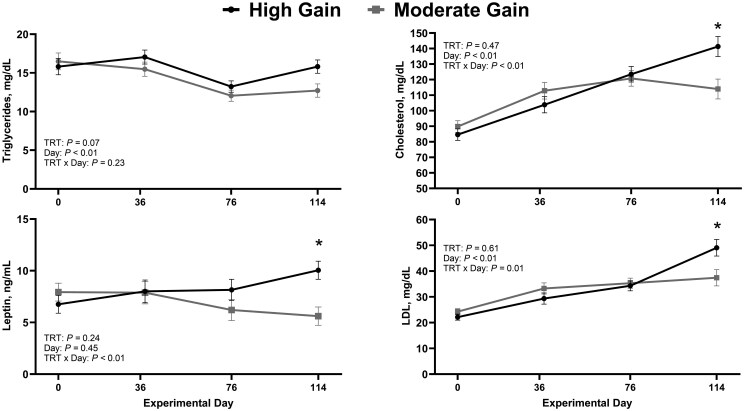
Temporal changes in systemic markers of positive energy balance based on dietary treatments (*n* = 44 bulls). Moderate Gain (MG): diet formulated to promote an average daily gain of 1.22 kg/d (*n* = 4 pens). High Gain (HG): diet formulated to promote an average daily gain of 1.81 kg/d (*n* = 4 pens). Dietary regimen was superimposed for 114 days. LDL: Low-density lipoprotein. ^*^Represent statistical differences in least square means between treatment within day (*P *≤ 0.05).

Results for metabolic markers associated with positive energy balance, including circulating concentrations of triglycerides, cholesterol, leptin, and LDL, are summarized in Figure 2. Bulls in the HG treatment tended (*P *= 0.07) to have greater circulating concentrations of triglycerides compared with bulls in MG treatment. An effect of day was also observed (*P *< 0.01), where circulating concentrations of triglycerides were decreased (*P *< 0.01) on day 76 compared with days 0 and 36. Moreover, a treatment × day interaction (*P *≤ 0.01) was observed for circulating concentrations of cholesterol, leptin, and LDL. Plasma concentrations of cholesterol, leptin, and LDL were not different (*P *≥ 0.19) between treatments on days 0, 36, and 76; however, HG bulls had greater (*P *≤ 0.01) circulating concentrations of cholesterol, leptin, and HDL on day 114 compared with MG bulls.

**Figure 3.**
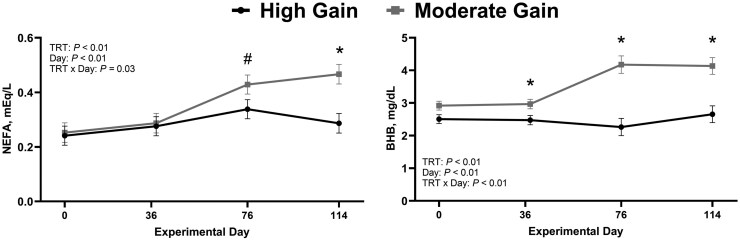
Impact of treatment on systemic markers of negative energy balance (*n* = 44 bulls). Moderate Gain (MG): diet formulated to promote an average daily gain of 1.22 kg/d (*n* = 4 pens). High Gain (HG): diet formulated to promote an average daily gain of 1.81 kg/d (*n* = 4 pens). Dietary regimen was superimposed for 114 days. Dark and gray lines represent HG and MG bulls, respectively. BHB: beta-hydroxybutirate. NEFA: non-esterified fatty acids. ^*^Represent statistical differences in least square means between treatment within day (*P *≤ 0.05). ^#^Represent a tendency for differences in least square means between treatment within day (*P ≤ *0.10).

Circulating concentrations of blood metabolites associated with catabolism are summarized in Figure 3. A treatment × day interaction (*P *≤ 0.03) was observed for both BHB and NEFA. Circulating concentrations of NEFA were similar (*P *≥ 0.28) between treatments on days 0 and 36 but tended to be decreased (*P *= 0.06) on day 76 and were decreased (*P *< 0.01) on day 114 in HG bulls compared with MG bulls. Similarly, circulating concentrations of BHB were similar between treatments on day 0 (*P *= 0.28) but were decreased (*P *≤ 0.03) on days 36, 76, and 114 in HG compared with MG bulls.

**Figure 4.**
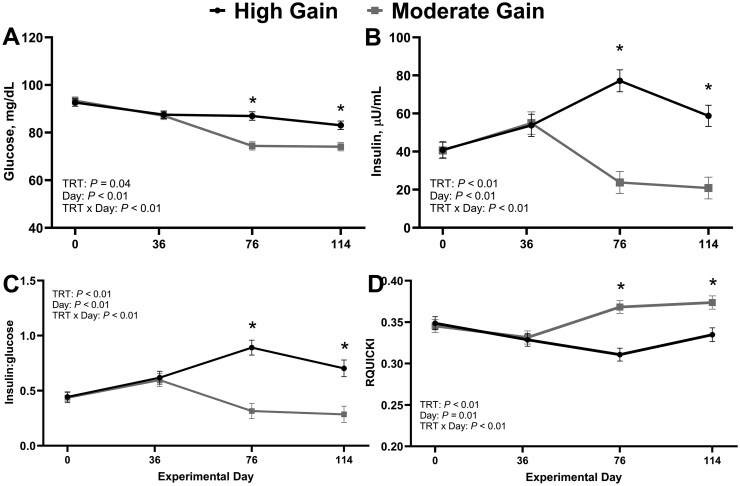
Impact of dietary treatment on estimates of insulin resistance (*n* = 44 bulls). Moderate Gain (MG): diet formulated to promote an average daily gain of 1.22 kg/d (*n* = 4 pens). High Gain (HG): diet formulated to promote an average daily gain of 1.81 kg/d (*n* = 4 pens). Dietary regimen was superimposed for 114 days. RQUICKI was estimated as described by Perseghin et al., (2001) using the following equation: RQUICKI = 1/[log(glucose) + log(insulin) + log(NEFA)]. TRT: treatment. ^*^Represent statistical differences in least square means between treatment within day (*P *≤ 0.05).

**Figure 5.**
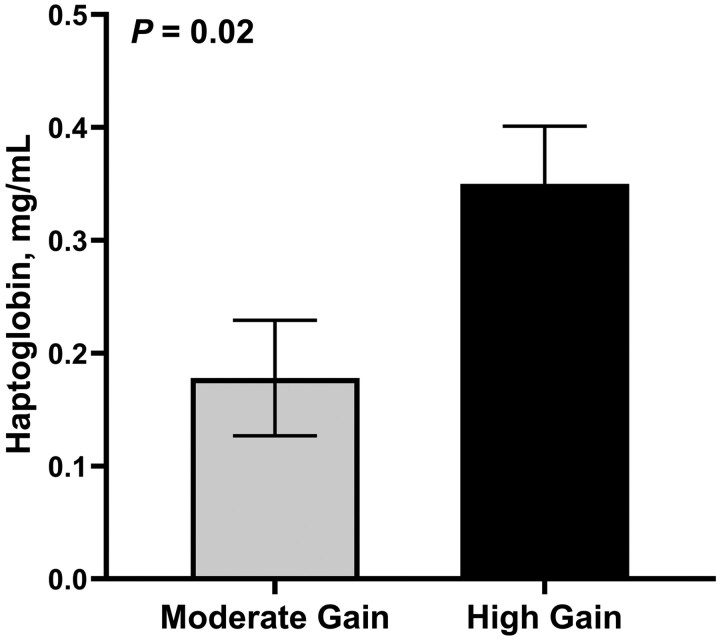
Effect of dietary treatment on circulating concentrations of haptoglobin on day 114 (*n* = 44 bulls). Moderate Gain (MG): diet formulated to promote an average daily gain of 1.22 kg/d (*n* = 4 pens). High Gain (HG): diet formulated to promote an average daily gain of 1.81 kg/d (*n* = 4 pens). Dietary regimen was superimposed for 114 days.

There was a treatment × day interaction (*P *< 0.01) for both circulating concentrations of glucose and insulin (Figure 4). Plasma glucose and insulin concentrations were similar (*P *≥ 0.73) between treatments on days 0 and 36 but were greater (*P *< 0.01) in HG compared with MG bulls on days 76 and 114. Similar results were observed for insulin: glucose ratio and RQUICKI index (treatment × day; *P *< 0.01; Figure 4). No differences between treatments were observed on days 0 and 36 (*P *≥ 0.72); however, HG bulls had increased (*P *< 0.01) insulin: glucose ration and decreased RQUICKI index values compared with MG bulls on both days 76 and 114. Plasma haptoglobin was only evaluated on day 114. Bulls in the HG treatment had a 2-fold increase in (*P *= 0.02) circulating concentrations of haptoglobin on day 114 compared with MG bulls (Figure 5).

### Impact of treatment on testicular and semen response variables

Chute-side scrotal traits and conventional spermiogram parameters are summarized in Table 2. Scrotal circumference at the end of the feeding period tended (*P *= 0.10) to be greater in HG compared with MG bulls; however, there were no differences between treatments on paired testis volume and scrotal skin thickness (*P *≥ 0.18). Ejaculate volume was decreased (*P *= 0.04) in HG bulls. Yet, there were no differences between treatments on sperm concentration, percentage of morphologically normal sperm, percentage of sperm with head, midpiece, and tail abnormalities, as well as no differences in systemic concentrations of testosterone on day 114 (*P *≥ 0.15). There were no differences (*P *= 0.31) in the percentage of ejaculates with ≥ 70% of morphologically normal sperm.

**Table 2.** Effect of dietary treatments on bull (*n* = 44) scrotal traits and conventional spermiogram analysis

	Treatment^1^			
	Moderate Gain	High Gain	SEM	*P*-value
**Scrotal circumference, cm**	36.8	38.0	0.49	0.10
**Paired testis volume, cm^3^**	618.4	769.9	78.10	0.18
**Scrotal skin thickness, mm**	2.90	2.84	0.10	0.69
**Ejaculate volume, mL**	5.0	4.0	0.33	0.04
**Concentration, millions/mL**	733.4	814.4	92.59	0.53
**Sperm morphology** ^2^				
** Normal cells, %**	68.0	64.6	3.20	0.45
** Head abnormalities, %**	18.1	23.3	2.76	0.15
** Midpiece abnormalities, %**	10.9	9.6	0.11	0.53
** Tail abnormalities, %**	1.0	1.2	0.20	0.50
**Ejaculates <70% normal cells, %**	35.6	46.0	7.14	0.31
**Testosterone, ng/mL**	1.91	2.25	0.61	0.69

1 Moderate Gain (MG): diet formulated to promote an average daily gain of 1.22 kg/d (*n* = 4 pens), or High Gain (HG): diet formulated to promote an average daily gain of 1.81 kg/d (*n* = 4 pens). Dietary regimen was superimposed for 114 days.

2 Semen morphology was evaluated according to Koziol and Armstrong (2018).

**Figure 6.**
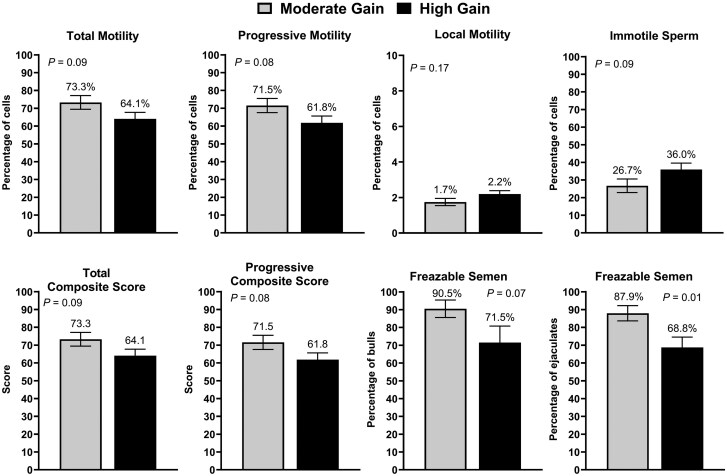
Computer assisted sperm analysis (CASA) of semen collected from bulls (*n* = 44) exposed to divergent planes of nutrition. Moderate Gain (MG): diet formulated to promote an average daily gain of 1.22 kg/d (*n* = 4 pens). High Gain (HG): diet formulated to promote an average daily gain of 1.81 kg/d (*n* = 4 pens). Dietary regimen was superimposed for 114 days.

Computer-assisted sperm analyses outcomes are summarized in Figure 6. The percentage of total motile and progressively motile sperm tended (*P *≤ 0.09) to be decreased in HG compared with MG bulls. Moreover, the percentage of immotile sperm tended to be greater in HG compared with MG bulls (*P *= 0.09). Total (*P *= 0.09) and progressive (*P *= 0.08) composite scores also tended to be decreased in HG compared with MG bulls. The percentage of bulls that met the progressive motility requirements for semen cryopreservation of ≥ 60% tended to be decreased in the HG compared with the MG treatment (*P *= 0.07). Similarly, fewer ejaculates met the motility requirements for cryopreservation in HG compared with MG bulls (*P *= 0.01). There were no differences (*P *≥ 0.17) in the percentage of local motile (Figure 2), rapid motile (HG: 46.4 ± 3.64%; MG: 53.1 ± 3.82%), and slow motile sperm (HG: 14.9 ± 0.95%; MG: 16.8 ± 0.99%) between treatments. Scrotal skin surface temperature was not altered in the top, middle, and bottom regions of the scrotum (*P *≥ 0.91). Moreover, there were no differences between treatments in temperature gradient between the top and bottom portions of the scrotum (*P *= 0.93; Supplementary Table S2—see online supplementary material).

**Figure 7.**
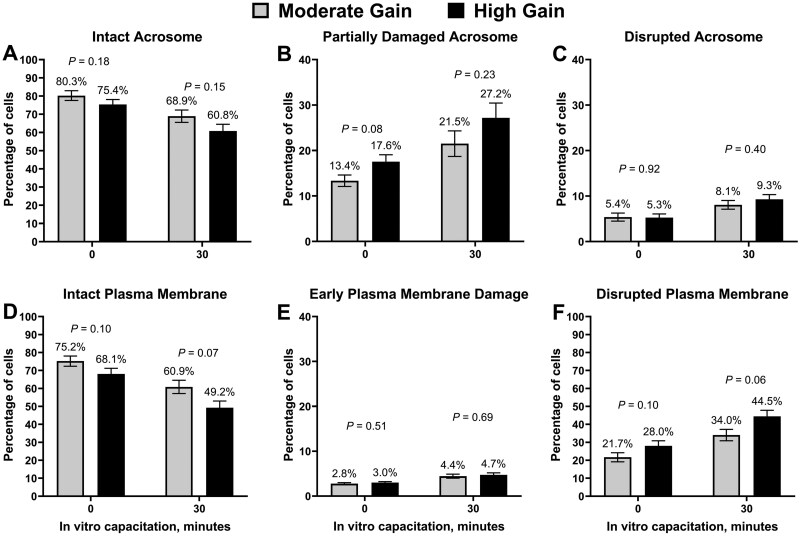
Image-based flow cytometry estimates of sperm acrosome and plasma membrane integrity from bulls (*n* = 44) exposed to divergent planes of nutrition. Moderate Gain (MG): diet formulated to promote an average daily gain of 1.22 kg/d (*n* = 4 pens). High Gain (HG): diet formulated to promote an average daily gain of 1.81 kg/d (*n* = 4 pens). Dietary regimen was superimposed for 114 days. Acrosome and plasma membrane integrity were evaluated at 0 and 30 minutes of in vitro capacitation as previously described (Zoca et al., 2023).

Impact of treatment on acrosome and plasma membrane integrity are summarized in Figure 7. There was no impact of treatment on the percentage of sperm classified as having intact or disrupted acrosome at both 0 and 30 minutes of IVC (*P *≥ 0.15). In contrast, HG bulls tended to have a greater percentage of sperm with partial acrosome damage at 0 (*P *= 0.08) but not at 30 minutes of IVC (*P *= 0.23). Bulls in the HG treatment tended to have a decrease (*P *≤ 0.10) in the percentage of sperm with intact plasma membrane at 0 and 30 minutes of IVC compared with MG bulls. Although there were no differences in the percentage of sperm with early plasma membrane damage (*P *≥ 0.51), bulls in the HG treatment tended to have a greater percentage of sperm with disrupted membrane at 0 (*P *= 0.10) and 30 minutes of IVC (*P *= 0.06). There were no differences (*P *= 0.74) between treatments in the percentage of sperm with DNA fragmentation (HG: 3.2 ± 0.55%; MG: 2.9 ± 0.55%).

## Discussion

Results reported herein indicate that the proposed model of bull overnutrition successfully resulted in an obese-like phenotype characterized by increased rates of body weight gain and adiposity. Treatments utilized in the present study promoted a 0.8 kg difference in average daily gain between treatments that resulted in a 15.9% increase in body weight in HG compared with MG bulls at the end of the feeding regimen. Average subcutaneous backfat thickness in HG treatment bulls was 1.2 cm, which was 92.8% greater than MG bulls. Notably, the magnitude of average daily gain and carcass adiposity achieved in HG bulls at the end of the feeding period is consistent with those observed in bull development programs in the beef industry (Henley et al., 2020; Smith et al., 2025). This similarity underscores the translational relevance of the present model and indicates that the responses to the dietary treatments observed in this study reflect conditions commonly encountered in bull development programs in the beef cattle industry.

To describe the systemic response of bulls to the dietary treatments utilized herein, multiple metabolic and endocrine markers were evaluated. Clear changes in metabolic indicators were observed, reinforcing the effectiveness of the proposed diets at eliciting a systemic response to proposed treatments. More specifically, HG bulls experienced an increase in circulating concentrations of insulin, glucose, triglycerides, cholesterol, LDL, and leptin, which are indicators of a positive energy balance (Sales et al., 2015; Sheehy et al., 2017). Furthermore, circulating concentrations of NEFA and BHB, which are indicators of negative energy balance in cattle (Richards et al., 1989; Fontes et al., 2021), were decreased in HG bulls. Dietary treatments also altered estimates of insulin resistance. Bulls in the HG treatment had greater insulin: glucose ratio and decreased RQUICKI values on days 76 and 114, indicating that HG bulls were experiencing greater insulin resistance compared with MG bulls. These findings align with previous reports in finishing cattle demonstrating reduced insulin sensitivity at the end of the finishing period relative to the beginning (Radunz et al., 2012; Kneeskern et al., 2016; Sousa et al., 2022).

Insulin resistance and adipose tissue dysfunction in humans is associated with pro-inflammatory adipokine secretions that contribute to the pathophysiology of metabolic syndrome (Vandanmagsar et al., 2011; Neeland et al., 2024). Haptoglobin is a glycoprotein synthesized in the liver and adipose tissue that participates in the acute-phase response and is commonly used as a systemic marker of inflammation in cattle (Young et al., 1996; Saremi et al., 2012; Cooke and Arthington, 2013; Pickett et al., 2024). In mice and humans, adipose tissue mass is associated with increased expression of haptoglobin in adipocytes, and increased circulating concentrations of haptoglobin is positively associated with body mass index (Nascimento et al., 2004; Chiellini 2004; Lisi et al., 2011). For these reasons, plasma haptoglobin concentrations were quantified in the present study. Circulating concentrations of haptoglobin at the end of the feeding period were 2-fold greater in HG compared with MG bulls, suggesting a proinflammatory response to the dietary treatment in HG bulls. Haptoglobin expression in bovine adipose tissue has been shown to increase in response to proinflammatory stimuli, such as TNF-α and lipopolysaccharide (Soliman et al., 2015), supporting the idea that adipose tissue can contribute to circulating haptoglobin concentrations under inflammatory conditions. Although studies in humans and rodents demonstrate a positive association between adiposity and haptoglobin expression (Nascimento et al., 2004; Chiellini 2004), comparable evidence in cattle remains limited. Therefore, while the greater haptoglobin concentrations observed in HG bulls may reflect an inflammatory response associated with sustained overnutrition, further research is needed to determine whether increased adiposity directly contributes to elevated haptoglobin in beef cattle.

Overnutrition resulted in subtle changes in semen quality in the present study. More specifically, bulls in the HG treatment tended to have decreased total and progressive sperm motility based on CASA. Additionally, a lesser percentage of ejaculates were classified as eligible for cryopreservation (progressive motility ≥ 60%) when compared with MG bulls. In an observational study evaluating the relationship between subcutaneous backfat thickness and sperm morphology in young beef bulls, Smith et al. (2025) reported a decrease in the percentage of morphologically normal sperm and an increase in the percentage of primary and secondary abnormalities in bulls with excessive subcutaneous backfat (approximately 1.2 cm). Moreover, a greater proportion of bulls in the top 20% of the population for subcutaneous backfat thickness were classified as deferred in their first breeding soundness examination when compared with bulls with less subcutaneous backfat (Smith et al., 2025). Sperm plasma membrane integrity was also influenced by dietary treatments in the present study. Bulls in the HG treatment tended to have a decreased percentage of sperm classified as having intact plasma membrane compared with MG bulls after both 0 and 30 minutes of IVC. Previous studies have also reported a decrease in the percentage of sperm with intact plasma membrane in bulls with reduced fertility (Anzar et al., 2002; Januskauskas et al., 2003; Zoca et al., 2020; 2023) and mature bulls exposed to overnutrition (Seekford et al., 2023).

Consequences of bull overnutrition to sperm fertilization ability were only evaluated indirectly through cleavage rates during in vitro embryo production (IVP; Seekford et al., 2023; Tariq et al., 2025). Seekford et al. (2023) observed no differences in cleavage rate of putative zygotes when semen from mature bulls exposed to overnutrition were utilized during IVP. Alternatively, Tariq et al. (2025) observed a decrease in the proportion of putative zygotes that successfully cleaved within 3 days after in vitro fertilization in bulls exposed to overnutrition. Interestingly, semen from bulls exposed to overnutrition had decreased IVP and resulted in embryos with increased cytoplasmic fragmentation at the 4-cell stage, increased inner cell mass and trophectoderm cell apoptosis at the blastocyst stage, and reduced number of trophectoderm cells (Seekford et al., 2023; Tariq et al., 2025). Collectively, these results indicate that although the young sire overnutrition model utilized in the present study only resulted in subtle changes in semen quality estimates, these changes can have significant consequences to the early embryonic development in the bovine (Seekford et al., 2023; Tariq et al., 2025).

Increased body fat deposition in bulls is positively associated with fat accumulation in the vascular cone region (Brito et al., 2012). Moreover, experimentally insulating the scrotum or the vascular cone region of the scrotum increases the percentage of morphologically abnormal sperm in the ejaculate, suggesting that impaired testicular thermoregulation might explain decreases in semen quality observed in bulls exposed to overnutrition (Barth and Bowman, 1994; Kastelic et al., 2018). In the present study, scrotal circumference tended to be 1.2 cm greater in HG compared with MG bulls at the end of the feeding period, corroborating previous reports in the literature where beef bulls fed a high-energy diet for 168 days had greater scrotal circumference compared with bulls fed a predominantly forage-based diet (Coulter et al., 1997). Interestingly, while some studies observed a decrease in the scrotal surface temperature gradient between the top and bottom portions of the scrotum (Coulter et al., 1997; Kastelic et al., 2018), no differences were observed in the present study. The lack of differences in the scrotal surface temperature suggests that the changes in sperm parameters observed herein were likely not driven by major changes in testicular thermoregulation. It is important to note that testicular infrared thermography images were collected only once and under conditions that did not induce heat stress. Although no differences in testicular thermoregulation were detected under these conditions, it is reasonable to speculate that potential impairments associated with excessive fat accumulation in the vascular cone may become more evident under heat stress scenarios. Adipokine signaling has been shown to disrupt Sertoli cell function, elevate testicular oxidative stress, and disrupt spermatogenesis (Li et al., 2006; Zhang et al., 2014; Feng et al., 2025), aligning with the results observed in the present study and providing a plausible mechanism by which overnutrition impaired semen characteristics in the present study. Yet, further research is required to test this hypothesis in the bovine using overnutrition models that replicate the magnitude of overnutrition observed in the beef cattle industry.

In summary, exposing growing beef bulls to overnutrition during development resulted in increased body adiposity and greater circulating concentrations of insulin, glucose, triglycerides, cholesterol, LDL, and leptin at the end of day 114 of the experiment. Bulls exposed to overnutrition had greater insulin resistance and increased circulating concentrations of haptoglobin. These responses to overnutrition were accompanied by subtle decreases in estimates of semen quality using both CASA and image-based flow cytometry. Although these changes in semen quality estimates are subtle, recent research indicates that overnutrition in bulls can negatively impact early embryonic development, highlighting the potential contributions of paternal diet to post-fertilization events and pregnancy establishment in cattle.

## Supplementary Material

skag004_Supplementary_Data

## Abbreviations: :

BHB: beta-hydroxybutyrate
CASA: computer assisted sperm analysis
ddH_2_O: double-distilled water
HG: high gain
IVC: in vitro capacitation
IVP: in vitro embryo production
LDL: low-density lipoproteins
LMA: longissimus dorsi area
MG: moderate gain
NEFA: non-esterified fatty acids
PI: propidium iodide
PBS: phosphate buffer saline
PNA: peanut agglutinin
RQUICKI: revised quantitative insulin sensitivity check index
