# Supplementary material for: Overnutrition induced metabolic dysregulation and partially decreased semen quality in young beef bulls
Source: J Anim Sci. 2026 Jan 12;104:skag004. doi: 10.1093/jas/skag004 (PMC12932944; doi:10.1093/jas/skag004)
Supplement: skag004_Supplementary_Data [file skag004_supplementary_data.zip › SUPPLEMENTARY_MATERIALS.docx]

**SUPPLEMENTARY MATERIALS**

**Supplementary Figure 1**. Representative image of scrotal surface temperature estimations using thermal imaging. Scrotal surface temperature was estimated in the top and bottom portions of the scrotum, as well as over each testis. Top portion represented the area dorsal to aspect of each testis, whereas the bottom portion was selected approximately 1.5 cm above the ventral bottom of the scrotum. Middle area represented the area between the top and bottom portion of the scrotum (left and right areas 1, 2, and 3). Scrotal surface temperature gradient was estimated by subtracting the average temperature of the bottom of the scrotum from the average temperature of the top of the scrotum.

|  | Treatment | |
| --- | --- | --- |
|  | Moderate Gain | High Gain |
| Ingredients, % (as-fed basis) |  |  |
| Steam flaked corn | 28.0 | 40.0 |
| Corn gluten feed | 20.0 | 25.0 |
| Soybean hulls | 11.0 | 14.0 |
| Peanut hulls | 15.0 | 0.0 |
| Cottonseed hulls | 15.0 | 13.0 |
| Soybean meal | 8.0 | 5.0 |
| Molasses | 2.0 | 2.0 |
| Limestone | 1.0 | 1.0 |
| Salt | 1.0 | 1.0 |
| Nutritive profile |  |  |
| Dry matter (DM), % | 87.8 | 87.3 |
| Crude protein, % of DM | 13.2 | 14.3 |
| NDF, % of DM | 50.6 | 35.8 |
| ADF, % of DM | 32.5 | 19.4 |
| Total digestible nutrients, % of DM | 56.4 | 68.9 |
| NE_m_, MCal/kg^2^ | 1.21 | 1.72 |
| NE_g_, MCal/kg^3^ | 0.64 | 1.10 |

**Supplementary Table 1**. Diet composition and nutrient analysis of dietary treatments.

^1^Analyzed by a commercial laboratory using wet chemistry package (Cumberland Valley Analytical Services, Waynesboro, PA, USA).

^2^Net energy for maintenance.

^3^Net energy for growth.

**Supplementary Table 2**. Bull (n = 44) scrotal surface temperature determined using thermal imaging.

|  | Treatment^1^ | |  |  |
| --- | --- | --- | --- | --- |
|  | Moderate Gain | High Gain | SEM | *P*-value |
| Scrotal surface temperature, ℃^2^ |  |  |  |  |
| Top | 36.9 | 36.9 | 0.19 | 0.94 |
| Middle | 35.0 | 35.0 | 0.14 | 0.97 |
| Bottom | 32.6 | 32.6 | 0.26 | 0.91 |
| Gradient | 4.3 | 4.4 | 0.29 | 0.93 |

^1^Moderate Gain (MG): diet formulated to promote an average daily gain of 1.22 kg/d (n = 4 pens), or 2) High Gain (HG): diet formulated to promote an average daily gain of 1.81 kg/d (n = 4 pens). Dietary regimen was superimposed for 114 days.

^2^Top scrotal region represented the area dorsal to aspect of each testis, whereas the bottom portion was selected approximately 1.5 cm above the ventral bottom of the scrotum. Middle area represented the area between the top and bottom portion of the scrotum (Supplementary Figure 1). Scrotal surface temperature gradient was estimated by subtracting the average surface temperature of the bottom of the scrotum from the average temperature of the top of the scrotum.
