# Supplementary figures and images for: Overnutrition induced metabolic dysregulation and partially decreased semen quality in young beef bulls
Source: J Anim Sci. 2026 Jan 12;104:skag004. doi: 10.1093/jas/skag004 (PMC12932944; doi:10.1093/jas/skag004)

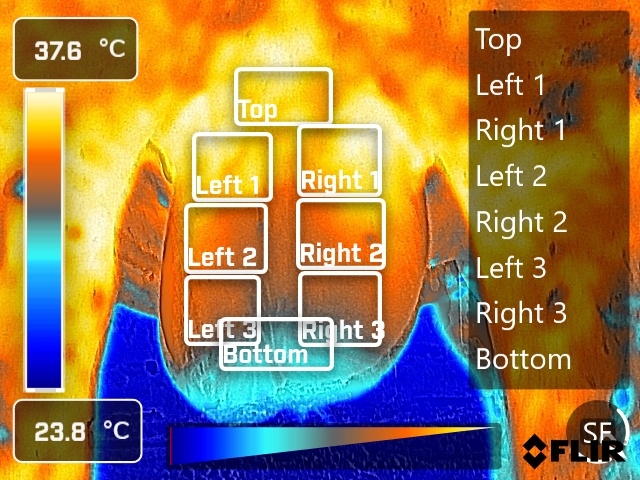

Supplement: skag004_Supplementary_Data [file skag004_supplementary_data.zip › Supplmentary_Figure_1.jpg]
